# Evidence base for point-of-care ultrasound (POCUS) for diagnosis of skull fractures in children: a systematic review and meta-analysis

**DOI:** 10.1136/emermed-2020-209887

**Published:** 2020-12-03

**Authors:** Georgios Alexandridis, Eva W Verschuuren, Arthur V Rosendaal, Danny A Kanhai

**Affiliations:** 1 Emergency Medicine, Erasmus Medical Center, Rotterdam, Zuid-Holland, The Netherlands; 2 Emergency Medicine, Franciscus Gasthuis en Vlietland, Rotterdam, Zuid-Holland, The Netherlands; 3 Pediatrics, Franciscus Gasthuis en Vlietland, Rotterdam, Zuid-Holland, The Netherlands

**Keywords:** imaging, ultrasound, imaging, CT/MRI, Trauma, head, paediatrics, paediatric injury, paediatrics, paediatric emergency medicine

## Abstract

**Background:**

Blunt head trauma is a common presentation to emergency departments (EDs). Identifying skull fractures in children is important as they are known factor of risk for traumatic brain injury (TBI). Currently, CT is the reference standard for diagnosing skull fractures and TBIs in children. Identifying skull fractures with point-of-care ultrasound (POCUS) may help risk-stratify children for TBI following blunt trauma. The purpose of this study is to evaluate the sensitivity, specificity, positive predictive value and negative predictive value of POCUS in identifying skull fractures in children.

**Methods:**

A systematic search was performed on 17 July 2020 in Ovid Medline, Cochrane Library, Google Scholar, Web of Science and Embase. Prospective studies reporting skull fractures diagnosed with ultrasound in children younger than 18 years due to blunt head injury were included. Studies that did not confirm the fracture with CT were excluded. The quality of studies was evaluated using the QUADAS-2 tool. Data were extracted from the eligible studies to calculate outcomes such as sensitivity and specificity; when possible overall outcomes were calculated.

**Results:**

Seven studies were included. All eligible studies included patients for whom the decision to perform a CT scan was made in advance. Overall, the included studies demonstrated low risk of bias or had minor concerns regarding risk of bias. The pooled data (n=925) demonstrated a sensitivity of 91%, specificity of 96%, positive predictive value of 88% and negative predictive value of 97%.

**Conclusion:**

The included studies demonstrate minor methodological limitations. Overall, the evidence suggests that POCUS is a valid option for diagnosing skull fractures in children visiting the ED after blunt head injury.

**Key messages**

**What is already known on this subject**

- Skull fractures are associated with traumatic brain injury; subsequently, clinical decision algorithms have been developed for head injured children that incorporate clinical suspicion for skull fractures.
- CT is the reference standard for diagnosing skull fractures and traumatic brain injuries; however, CT exposes children to radiation.
- Point-of-care ultrasound is a rapid and non-invasive diagnostic tool that has been used to identify skull fractures.

**What this study adds**

- This systematic review reveals that the included studies have minor methodological limitations and that point-of-care ultrasound has a high sensitivity and specificity to identify skull fractures in children with blunt head injuries.
- Point-of care-ultrasound could serve as an adjunct to increase the accuracy of clinical decision rules regarding using CT scans after head trauma in children; future research is necessary to define this role.

## Introduction

Head injuries in children are a common presentation to emergency departments (EDs) point-of-care ultrasounds (POCUS). The Centers for Disease Control and Prevention in the USA reported 749 000 ED visits for injury to the head in children under the age of 15 years old in 2016.^1^ Up to 8% of children aged 3–17 years old have had a significant head injury in their lifetime,^2–6^ and it is one of the leading causes of mortality in children worldwide.^3^ Severely head-injured children are in general easily identified and treated. However, children who appear to have a low risk for traumatic brain injury (TBI) may still have clinically important injury.^2^ POCUS may have a potential role in the evaluation of low-risk paediatric patients, to identify skull fractures and subsequent TBIs.

Clinical decision algorithms have been developed to identify head-injured children with TBI (eg, Pediatric Emergency Care Applied Research Network [PECARN], Canadian Assessment of Tomography for Childhood Head injury [CATCH], National Institute for Health and Care Excellence [NICE] Head Injury guideline or Children’s Head injury ALgorithm for prediction of Clinically Important Events [CHALICE]).^2 7–9^ Most of the clinical decision algorithms include clinical suspicion of a skull fracture because the presence of skull fracture increases the likelihood of an intracranial injury fourfold.^2 4 8 10–13^ Furthermore, TBI in the absence of skull fracture is rare in the paediatric population.^14^ The reported rate of skull fracture in children with blunt head trauma ranges from 16% to 63%.^11 14 15^ In addition, infants with skull fractures rarely present without local signs of head injury on physical examination.^15 16^ The diagnostic standard tool to detect TBI is CT. CT exposes children to radiation that may increase lifetime risk of malignancy.^15–19^ Also, it has been demonstrated that low doses of ionising radiation to the brain in infancy may influence cognitive ability in adulthood.^20^ Occasionally sedation is needed during the diagnostic study to minimise motion artefacts; sedation is also associated with the risk of adverse respiratory events including desaturation and the need for airway intervention.^21^ The use of CT scanning also adds to the costs of healthcare.^22^

‘Mild head injury’ patients are, according to the Head Injury Severity Scale classification, initially conscious at first assessment (GCS score 14–15), may have had a brief loss of consciousness or amnesia, but do not have any focal neurological deficits on admission.^23^ In mild head-injured children ultrasound might be utilised as a diagnostic tool for identifying skull fracture in children that may otherwise undergo a head CT.^24–26^

POCUS has become an integral part of emergency medicine practice. It is a rapid, non-invasive and inexpensive diagnostic tool that does not carry the risk of radiation in comparison to CT.^27^ Furthermore, in children who are more anxious and easily overstimulated, POCUS may be used by the attending physician at the bedside.

Identification of skull fractures in children is clinically relevant because these fractures may need surgical intervention irrespective of the presence of TBI.^28^ Skull fractures could be associated with non-accidental trauma, and physicians should always consider this potential association.^29^ In addition, skull fractures may need follow-up because linear fractures may predispose children to uncommon but serious complications such as expanding fractures or leptomeningeal cysts.^30 31^

Clinicians must weigh the risk of missing a clinically important skull fracture and potentially associated TBI and the risks associated with performing a CT. As children with TBI can be asymptomatic, it remains challenging to successfully identify children at very low risk for TBI and safely manage them without performing a CT scan. However, if it is possible to rule out skull fractures utilising POCUS with high sensitivity, then it is less likely that there is an associated TBI. This may reduce the number of CT scans performed. Also, in hospitals where CT is not readily available POCUS may be helpful in risk stratification and subsequent transport to another facility.

This systematic review evaluates the test characteristics of POCUS in identifying skull fractures in children that present with blunt head injury in the ED.

## Materials and methods

The Preferred Reporting Items for Systematic Reviews and Meta-Analyses guideline was used to conduct this review.^32^ Prior to performing this review, a protocol was developed (see online supplemental A). Consensus was reached among all authors on search syntax, inclusion and exclusion criteria, and the criteria for the assessment of validity and relevance in the identified articles.

10.1136/emermed-2020-209887.supp1 Supplementary data

### Search strategy

A search was conducted on 17 July 2020 using the search engines Ovid Medline, Cochrane Library, Google Scholar, Web of Science and Embase. The search syntax encompassed ‘skull’, ‘ultrasound’, ‘child’ and ‘fracture’ (including their respective synonyms). Online supplemental B contains an overview of the complete search syntax. All articles which met the search terms were exported from the search engines to EndNote X9 (Clarivate Analytics, Philadelphia, Pennsylvania, USA).

10.1136/emermed-2020-209887.supp2 Supplementary data

### Selection

**Figure 1:**
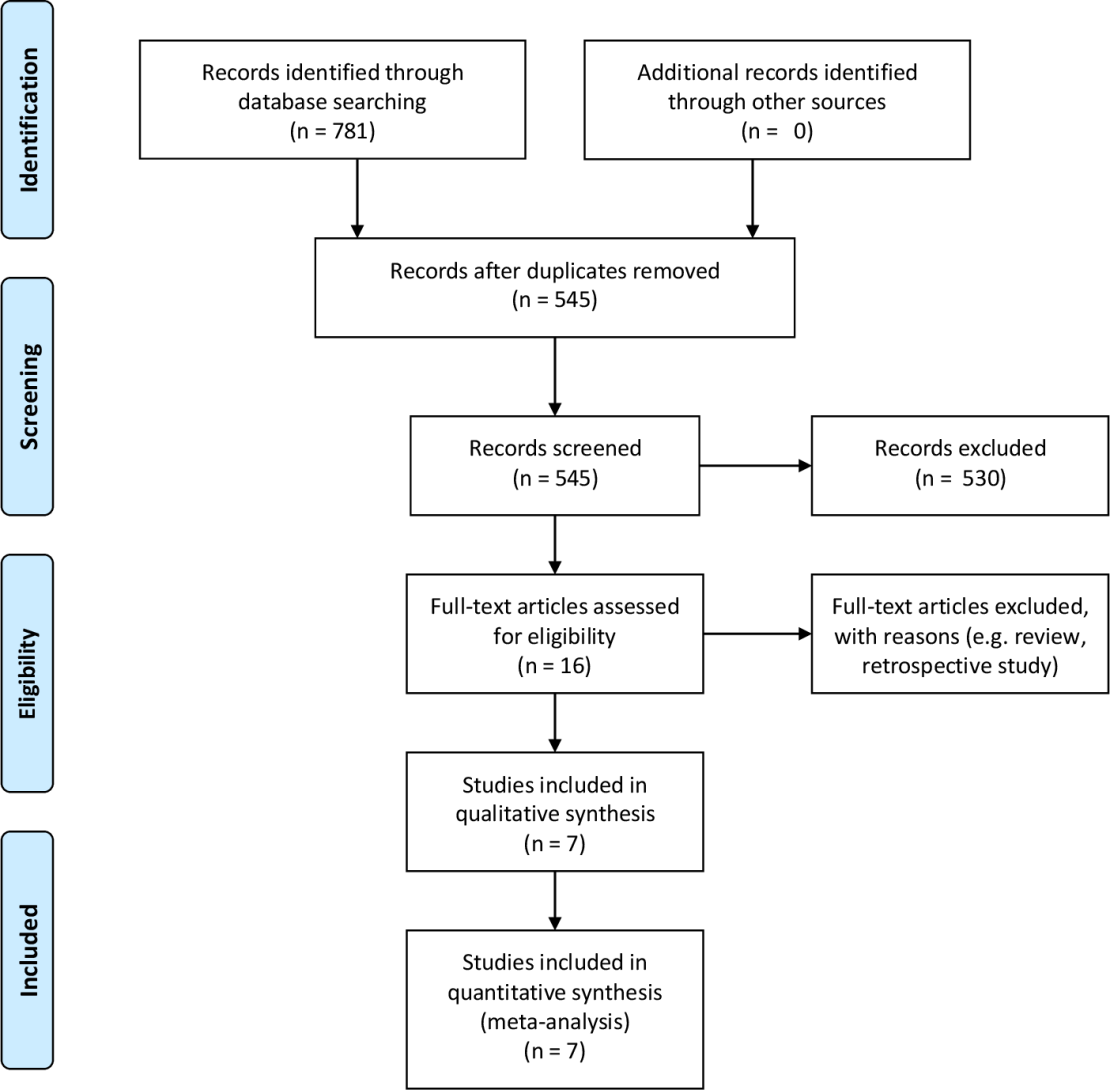
Flow-chart of search strategy and selection

Duplicates were excluded. We analysed all articles evaluating the outcome ‘skull fracture’. Prospective studies written in English, German, French and Dutch that studied children with blunt head injury who visited the ED were included. Based on the inclusion criteria (figure 1), two reviewers (GA and EV) independently screened the titles and later the abstracts of eligible articles. Review articles, conference abstracts and case reports were excluded. The full text of the remaining articles was screened independently by two reviewers (EV and GA). Additionally, references in review articles were screened using the same criteria.

### Critical appraisal

The quality of the individual studies was evaluated using the Quality Assessment of Diagnostic Accuracy Studies 2 (QUADAS-2) tool.^33^ This tool aids in evaluating the quality of primary diagnostic accuracy studies. It consists of four key domains; patient selection, index test, reference standard and flow and timing. Each domain is assessed in terms of the risk of bias, as ‘low’, ‘high’ or ‘unclear’. Signalling questions are provided to help in judgement on the risk of bias. An overall judgement per study was given. If a study is judged as ‘low’ on all domains relating to validity than the overall judgement for that particular study was ‘low risk of bias’; when one or more domains in validity was judged as ‘unclear’ or ‘high’ than that particular study was considered to be ‘at risk of bias’.^33^

Three authors (GA, EV and AVR) independently evaluated the quality of the eligible studies. In case of disagreements in the critical appraisal, consensus was reached through discussion.

### Data extraction and statistical analysis

Two authors extracted data separately from the eligible studies (EV and GA). The following data were extracted: year of publication, study characteristics, baseline population characteristics, country and data on the test results. When possible, the data of the studies was pooled to calculate overall characteristics. A fixed-effect model was used to compute pooled sensitivity and specificity. The homogeneity of the studies was determined with visual inspection; the homogeneity in age and trauma mechanism were analysed. I^2^ was used to evaluate the between-study heterogeneity throughout the random-effects meta-analysis; Q-test was used to determine whether the heterogeneity was statistically significant. Publication bias was assessed through Egger’s regression test. Comprehensive Meta-Analysis V.3 (Biostat, Englewood, New Jersey, USA) was used to perform all statistical testing.

## Results

### Search strategy and selection

The search resulted in 781 articles. After exclusion of duplicates 545 articles remained for title and abstract screening. Full text and reference assessment was completed for 16 articles; afterwards seven articles were excluded because they were reviews and two because they were retrospective studies (figure 1). We manually checked the references of the reviews; our search had identified all studies referenced in these reviews.

### Critical appraisal

Seven studies were included; two studies had a low risk of bias.^34 35^ Five studies were at risk of bias in the domain ‘patient selection’.^36–40^ One study was at risk of bias in the domain ‘flow and timing’ because the time interval between POCUS and CT was unclear.^36^ An overview is presented in table 1.

**Table 1:** Critical appraisal

	Study design	Relevance (applicability)		Validity (risk of bias)					
		Domain	Reference standard	Patient selection	Index test	Reference standard	Flow and timing	Remarks	Overall risk of bias
Weinberg^36^	Prospective observational study	Age <25 years, urban centre.	CT	High	Low	Low	Unclear	Not only skull	At risk of bias
								fractures were assessed.	
								Convenience	
								Sample.	
								Unclear interval	
								between POCUS and CT	
Riera^37^	Prospective observational study	Age <18 years,	CT	High	Low	Low	Low	Convenience sample	At risk of bias
		Tertiary care centre and trauma level one centre.							
Parri^35^	Prospective observational study	Age <18 years.	CT	Low	Low	Low	Low	–	Low risk of bias
Rabiner^38^	Prospective observational study	Age <21 years, trauma level two centre.	CT	High	Low	Low	Low	Convenience sample	At risk of bias
Choi^39^	Prospective observational study	Age <4 years, tertiary care centre and trauma level I centre.	CT	High	Low	Low	Low	Convenience sample	At risk of bias
Parri^40^	Multicentre prospective observational study	Age <2 years.	CT	High	Low	Low	Low	Convenience sample	At risk of bias
Masaeli^34^	Prospective cross-sectional study	Age <18 years, tertiary care centre.	CT	Low	Low	Low	Low	–	Low risk of bias

POCUS, point-of-care ultrasound.

All studies had a prospective observational study design and included patients with head trauma. In all eligible studies, the decision to perform a CT was made in advance.

### Data extraction

The results of data extraction are shown in tables 2 and 3.

**Table 2:** Study and population characteristics

	Study characteristics				Population characteristics		
	No of patients (N=)	Country	Training	Experience	Age	Trauma mechanism (%)	Incidence of fractures (%)
Weinberg^36^	21	USA	1 hour	NR	Median 13 y	NR	10
Riera^37^	40	USA	NR	1 mo to 10 y	Median 2 y (2 mo to 17 y)	NR	15
Parri^35^	55	Italy	1 hour	16 hours	Mean 5 y (2 mo to 14 y)	Fall (71)	64
Rabiner^38^	69	USA	1 hour	variable	Mean 7 y (7 days to 21 y)	NR	12
Choi^39^	87	South Korea	1 hour	variable	Mean 21 mo (2 mo to 48 mo)	Fall <0.9 m (67)	15
Parri^40^	115	Italy, USA	Two videos and skills	variable	Mean 8 mo (SD 6)	Fall from elevation (75)	77
Masaeli^34^	538	Iran	Theory and skills workshop	NR	Mean 6 y (SD 5, range 0–18 y)	Fall (44), motor vehicle accidents (15), other (41)	14

mo, months; NR, not reported; y, year.

**Table 3:** Results

	No of patients (N=)	Sensitivity	Specificity	Positive predictive value	Negative predictive value	False-positive	False negative	Risk of bias
		%	%	%	%	N=	N=	
		(95% CI)	(95% CI)	(95% CI)	(95% CI)			
High percentage of fractures								
Parri^35^	55	100 (88 to 100)	95 (73 to 100)	97 (84 to 100)	100 (79 to 100)	1: Non calcified suture ipsilateral to trauma	0	Low risk of bias
Parri^40^	115	91 (82 to 96)	85 (65 to 95)	95 (88 to 98)	74 (55 to 87)	4: Not reported	8: Fracture not underneath area that was imaged	At risk of bias
Low percentage of fractures								
Weinberg^36^^*^	21	100 (20 to 100)	100 (79 to 100)	100 (20 to 100)	100 (79 to 100)	0	0	At risk of bias
Riera^37^	40	67 (24 to 94)	97 (83 to 100)	80 (30 to 99)	94 (79 to 99)	1: Not reported	1: Not reported	At risk of bias
							1: No cooperation of patient	
Rabiner^38^^*^	69	88 (47 to 99)	97 (88 to 99)	78 (40 to 96)	98 (90 to 100)	1: Novice error	1: Fracture adjacent to haematoma in the area that was not imaged	At risk of bias
						1: CT no fracture, possibly false negative as the sonographers seem to be convinced that it truly was a fracture		
Choi^39^	87	77 (46 to 94)	100 (94 to 100)	100 (66 to 100)	96 (88 to 99)	0	2: Difficult evaluation in area of orbital wall and skull base	At risk of bias
							1: Fracture not in imaged area, adjacent to haematoma	
Masaeli^34^	538	92 (83 to 97)	96 (94 to 97)	79 (69 to 87)	99 (97 to 99)	19: Not reported	6: Not reported	Low risk of bias
Pooled data								
Overall pooled data	925	91 (87 to 94)	96 (94 to 97)	88 (84 to 92)	97 (95 to 98)	27	20	
Pooled data A	170	93 (87 to 97)	89 (76 to 96)	96 (90 to 98)	84 (70 to 92)	5	8	
Pooled data B	755	89 (81 to 94)	97 (95 to 98)	81 (73 to 88)	98 (97 to 99)	22	12	
Pooled data C	720	92 (85 to 96)	96 (95 to 98)	85 (78 to 90)	98 (97 to 99)	21	11	
Pooled data D	835	91 (87 to 95)	96 (94 to 97)	88 (84 to 92)	97 (95 to 98)	25	19	

Pooled data A (studies with a high percentage in fractures): Parri^35^ and Parri^40^.

Pooled data B (studies with a low percentage in fractures): Weinberg^36^, Riera^37^, Rabiner^38^, Choi^39^ and Masaeli^34^.

Pooled data C (studies with a low percentage in fractures, excluded the studies that contained patients aged >18 years): Riera^37^, Choi^39^ and Masaeli^34^.

Pooled data D (all studies, excluded the studies that contained patients aged >18 years): Riera^37^, Parri^35^, Parri^40^, Choi^39^ and Masaeli^34^.

*Study that contained patients aged >18 years.

### Statistical analysis

Visual inspection showed that two studies had a substantially higher percentage of skull fractures^35 40^; subsequently, we divided the studies in two groups. In one group, the two studies with a high percentage of fractures were pooled and the other group contained the five studies with a low percentage of fractures. Furthermore, two studies included patients aged 18 years or older; subsequently, to assess the influence on outcome results a separate analysis was executed in which these two studies were excluded.^36 38^ Afterwards, the outcome results per group were calculated (see table 3). In the overall pooled data I^2^ was 32.14 and Q was 8.84. The Egger’s regression intercept did not demonstrate a publication bias (p=0.313).

### Patient characteristics

All studies included patients from the ED. The number of patients included in the studies ranged from 21 to 538. Three studies included patients up to 18 years old.^34 35 37^ The study of Parri *et al* 40 included children under the age of 2 and the study of Choi *et al* 39 children up to 4 years of age.^39 40^ One study included patients up to 21 years and another study up to 25 years old.^36 38^ Four studies reported a mean age between 8 months and 7 years.^35 38–40^ Weinberg *et al* 36 reported a median age of 13 years, and Riera and Chen^37^ a median of 2 years.

Four studies included patients with minor head injury (GCS 14 or 15).^34 35 39 40^ Two studies did not report the GCS.^36 37^ Masaeli *et al* 34 reported that 11% of the included children had a GCS lower than 15; this particular study excluded patients that had a GCS of 13 or lower.^34^ Rabiner *et al* 38 reported a GCS below 15 in 12% of the included patients.^38^

Trauma mechanism was described in four studies; in up to 75% of the patients the mechanism of trauma was a fall.^34 35 39 40^ The percentage of fractures per study ranged from 10% to 77%.

### Training and experience

In all studies ultrasound was performed by emergency physicians or fellows. Four studies reported 1-hour training focused on ultrasonography of the skull.^35 36 38 39^ In two studies the clinicians were trained with videos and skills training.^34 40^ In four studies the ultrasonographer had varying levels of experience.^37–40^ Two studies did not report the previous experience of the sonographers.^34 36^ One study reported that the sonographers had 16 hours of experience.^35^ Training and experience are summarised in table 2.

### Diagnostic accuracy

Sensitivity ranged from 67% to 100% and specificity from 85% to 100%. The seven studies demonstrated a weighted percentage of skull fractures of 25%. The overall pooled data (n=925) demonstrated a sensitivity of 91%, a specificity of 96%, a positive predictive value of 88% and a negative predictive value of 97% (see table 3).

**Figure 2:**
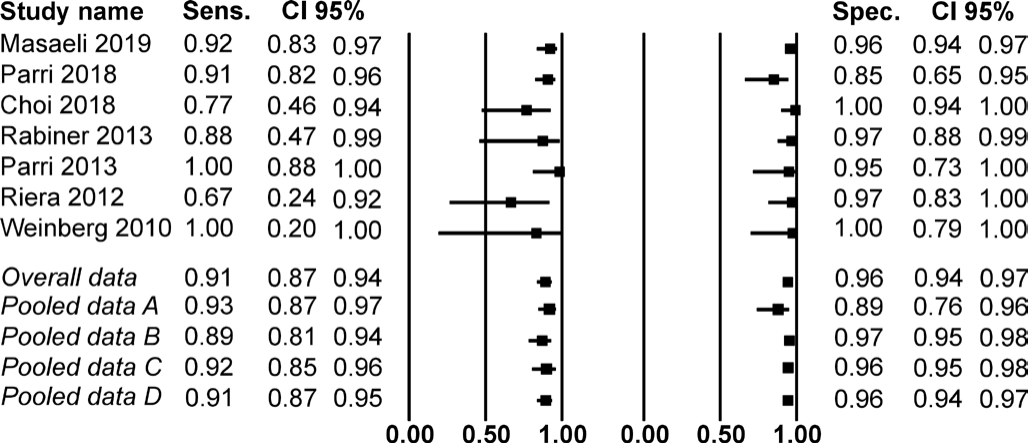


The pooled results of studies with a high percentage of fractures were similar to those of the studies with a low percentage of fractures (pooled data A and B in table 3). Furthermore, the results were similar whether those over 18 were included or excluded. (pooled data B, C, D and overall pooled data). To illustrate the similarities, we created a paired forest plot for sensitivity and specificity (see figure 2).

### False positive results

There were 27 false positive results (2.9% of all scans). In 24 of these cases, the cause was not reported. Parri *et al* 35 reported a false positive result due to a non-calcified ipsilateral suture. One case was the effect of a novice error, as the physician interpreted the ultrasound as positive, an expert sonographer interpreted the ultrasound as negative for fracture.^38^ Another case was interpreted during the initial POCUS imaging as positive for a fracture and during re-evaluation by an expert paediatric emergency physician sonographer as a minimally displaced skull fracture; however, this fracture was not visualised by the CT scan.^38^

### False negative results

Twenty false negative cases were reported (2.2% of all scans). The majority of these false negatives (n=10) were the consequence of the fracture being adjacent to a haematoma and not in the area that was imaged.

## Discussion

The aim of this study was to evaluate the test characteristics of POCUS in identifying skull fractures in children that presented in the ED with blunt head injury. The pooled data showed a high sensitivity, specificity and predictive values in the studies included. Our systematic review, therefore, demonstrates that POCUS of the skull can be useful in accurately identifying skull fractures in children with head injury.

While conducting this systematic review, another systematic review by Gordon *et al* 41 was published^41^; our findings are consistent with that review. Our review used a more extensive search, using more databases and including articles published in multiple languages. Subsequently, we identified an additional relevant study, which adds a substantial number of patients to our pooled data. Furthermore, additional subanalyses were performed to evaluate whether these subgroups (studies that included patients over the age of 18 years, and high vs low percentage of fractures per group) affected the results.

POCUS could serve as an adjunct to increase the accuracy of clinical decision rules regarding the use of CT scans after head trauma in children. POCUS is fast and may be performed at bedside, which provides benefits of rapid identification of skull fracture, that could help to prioritise patients. Furthermore, POCUS may help with the decision to transfer the patient to another facility. Moreover, no pharmacological sedation is needed for this diagnostic tool.

The identified studies in this review show possible limitations in applicability. First, all studies had varying experience in POCUS performers (see table 2). Because the interpretation of POCUS is operator dependent, this may have affected the diagnostic accuracy.^37^ Rabiner *et al* 38 reported that the interobserver agreement was κ=0.86 (95% CI 0.67 to 1.0). The other studies did not report the inter-observer agreement among physicians that performed POCUS.

Second, the range in percentage of fractures per study was wide (10%–77%); potentially affecting the test accuracy. Parri *et al* 35 and Parri *et al* 40 had a higher percentage of fractures. The younger age of the population studied may have influenced the incidence; children under the age of 2 tend to be at higher risk of skull fractures.^4 11^ Although there was overall a high sensitivity and specificity of the test, this pretest probability has to be for accounted for. The study of Choi *et al* 39 did not apply clinical decision rules and this factor possibly led to a lower number of fractures; this could also have occurred in the other studies with a relatively low number of fractures.^36–38^

Third, two studies included patients older than 18 years.^36 38^ For both studies, insufficient data regarding the proportion of patients aged 18 years or above was provided. However, it seems unlikely that a significant number of patients was older than 18 years because the reported mean age was 7 years in one study and in the other studies the reported median age 13 years; in addition, the subanalysis showed similar results. Hence, the influence seems to be minor.^36 38^

Fourth, many studies did not report the mechanism of injury; it seems likely that presence of a skull fracture may be associated with the mechanism. Furthermore, not all the studies reported GCS. It is unclear to what extent this influenced the results.

Finally, only two studies were multicentre.^34 40^ Three studies were conducted in a tertiary care centre, which may have influenced the population studied; in general, such centres receive more severe injured patients.^34 37 39 42 43^

Five of the seven studies were at risk of bias because they studied a convenience sample; this might lead to under-representation of subgroups.^36–40^

The study of Weinberg *et al* 36 has a remarkably high sensitivity and specificity of 100%. This may be explained due to the relatively small sample size of 21 patients that might overestimate the results. Also, the study is at risk of biases that might influence the results positively.

The study of Riera and Chen^37^ has a notable lower sensitivity for identifying skull fractures; this lower sensitivity was attributed to the lower number of skull fractures in the studied population.^37^ Several studies demonstrated that not all skull fractures were located beneath the haematoma; those studies only imaged the haematoma area with ultrasonography. By imaging a larger part of the skull these missed fractures may have been avoided.

Two ‘false negative’ fractures were located in a position where ultrasound evaluation is difficult, for example, orbital wall and skull base. It might be advised to reconsider imaging in children that present with a haematoma at sites that are more challenging to ultrasound or when interpretation of the ultrasound is difficult. It is key to minimise false negatives, because of the association of skull fractures with TBI. Perhaps with better ultrasonography techniques in addition to better training, false negatives may be avoided in the future. However, none of the studies reported adverse events in the cases with a missed fracture.

Suture planes can complicate the identification of fractures, and could lead to false positive results. However, evaluation of the contralateral anatomy or tracking suture lines to the fontanelle may aid in this, and knowledge of suture line anatomy is essential.^35 37 38^

Our search is limited to articles written in English, Dutch, German or French. During the abstract screening, none of the identified articles were excluded on language. The included studies all had positive results in favour of POCUS; it is possible that studies with other outcomes did not get published, therefore, creating publication bias. In any literature review, there is the possibility of publication bias; however, the Egger’s regression intercept did not demonstrate this particular bias.

This systematic review shows that POCUS proves to be a valid option for ruling out skull fractures in children visiting the ED after blunt head injury, although methodological limitations could be debated in several included studies.

We recommend consideration of POCUS in patients that meet no other major or multiple minor criteria for CT scan than a clinical suspected skull fracture, or when a skull fracture cannot be excluded; consequently, when no fracture is identified, a CT scan might not be needed. Therefore, to use POCUS to rule out a skull fracture could potentially reduce the utilisation of CT and unnecessary exposure of children to ionising radiation. The aim of POCUS is not to detect TBI, but it can identify skull fracture which is an important predictor of TBI.^44^

Future research is necessary to define the role of POCUS in risk stratification for TBI and applicability in clinical prediction rules. Furthermore, additional research is needed to define whether extending the imaged area with POCUS to not only the area of haematoma but also adjacent areas will further improve the results.

10.1136/emermed-2020-209887.supp3 Supplementary data
