# Supplementary material for: Evidence base for point-of-care ultrasound (POCUS) for diagnosis of skull fractures in children: a systematic review and meta-analysis
Source: Emerg Med J. 2020 Dec 3;39(1):30–6. doi: 10.1136/emermed-2020-209887 (PMC8717482; doi:10.1136/emermed-2020-209887)
Supplement: Supplementary data [file emermed-2020-209887supp001.pdf]

Title

The evidence base for point-of-care ultrasound (POCUS) for diagnosis of skull fractures in children: a systematic review

Registration

This review has not been registered

Authors

Eefje Verschuuren (corresponding author)

Department of Emergency Medicine, Franciscus Gasthuis & Vlietland

PO box 10900, Suite: SEH, 3004 BA Rotterdam, The Netherlands

Tel: +31 (0) 6 12549510; Eefje Verschuuren, e.verschuuren@franciscus.nl

Georgios Alexandridis

Franciscus Gasthuis & Vlietland

j.alexandridis@franciscus.nl

Arthur Rosendaal

Franciscus Gasthuis & Vlietland

a.rosendaal@franciscus.nl

Written in line with the PRISMA-P 2015 checklist [1]

Contributions:

EV and GA contribute equally in realizing the systematic review. A statistician was invited to evaluate the method of this review. An information specialist has been requested to create the search syntaxes. EV and GA performed the search, critical appraisal and extraction of data. EV and GA are first and second reviewer. AR is the third critical appraiser and companion in discussion. A paediatrician and a radiologist will be invited to thoroughly evaluate the review. All authors will contribute to data interpretation and article drafts.

Amendments

Important protocol amendments post registration will be recorded and included in dissemination.

Support

No sources of support or funding were provided for this review. EV and GA are emergency medicine residents-in-training. AR is an emergency medicine physician.

Introduction

Blunt head trauma is a common presentation to emergency departments (ED). Identifying skull fractures in children is important as they are a known risk factor for traumatic brain injury (TBI).[2-8] Currently, computer tomography (CT) scan is the reference standard for diagnosing skull fractures and TBIs in children. However, CT scans expose children to radiation that may increase lifetime risk of lethal malignancy[9-12] . Identifying skull fractures with point-of-care ultrasound (POCUS) may help risk-stratify children for TBI following non-penetrating trauma. The purpose of this study is to evaluate the sensitivity, specificity, negative predictive value and positive predictive value of POCUS in identifying skull fractures in children.

Objective

What is the sensitivity, specificity, negative predictive value and positive predictive value of POCUS in identifying skull fractures in children that present with blunt head injury in the emergency department?

Methods

The Preferred Reporting Items for Systematic Reviews and Meta-Analyses (PRISMA) guideline shall be used to conduct this review.[13] Prior to conducting this review consensus shall be reached among all authors on search syntax, criteria of inclusion and exclusion, and the criteria for the assessment of validity and relevance in the identified articles

Eligibility criteria

- Children aged <18 years with blunt head injury visiting the emergency department
- Ultrasound and CT used as means to diagnose skull fracture
- Prospective study
- Articles in English, German, French or Dutch

Information sources

The search will be conducted in the search engines Ovid Medline, Cochrane Library, Google Scholar, Web of Science and Embase. Only the first hundred results in Google Scholar will be used for the selection process.

Search strategy

The search syntax encompasses ‘skull’, ‘ultrasound’, and ‘fracture’ (including their respective synonyms). See Table 1 for complete search syntax.

Study records

Data management:

Data will be managed using EndNote X9, Clarivate Analytics, Philadelphia, USA.

Selection process

Our aim is to include all prospective studies written in English, German, French or Dutch that assessed children with blunt head injury and visited the emergency department for this. Firstly, all duplicates shall be excluded. Based on the inclusion criteria two reviewers (GA and EV) screen the titles and abstracts of the articles. We shall exclude articles that are reviews, conference abstracts or case reports. The full text of the remaining articles will be screened by two reviewers (EV and GA). From these articles and the identified reviews, all references shall be screened utilizing the same criteria.

Data collection process

Two authors shall extract data separately from the eligible studies (EV and GA).

Data items

The following data shall be extracted: year of publication, study characteristics, baseline population characteristics and data on the outcomes.

Risk of bias in individual studies

The quality of the individual studies shall be evaluated utilizing QUADAS-2.[14] Three authors (GA, EV and AR) will independently evaluate the quality of the eligible studies. The aim is to reach consensus among the critical appraisers through discussion.

Data Synthesis

Data will be presented in tabulated form to allow for semi qualitative comparison of; study sample, population characteristics, training and experience of physicians, trauma mechanism and incidence of fractures. Preferably the sensitivity, specificity, negative predictive value and positive predictive value will be reported; moreover, the false positive and false negatives cases will be reported.

Meta-analysis

If possible, the data of the studies will be pooled to calculate overall outcomes. The homogeneity of the studies will be determined with visual inspection; we shall analyze the homogeneity in age and trauma mechanism.

Meta-biases

To identify possible 'gray literature' we shall include a computerized search in Google Scholar.

Acknowledgements

We would like to acknowledge Wichor M. Bramer (biomedical information specialist) for his aid in creating the search syntax and performing the initial search; moreover, that he combined the initial identified articles and was kind enough to remove the duplicates. In addition, we would like to acknowledge Erwin Birnie (statistician) for evaluating the method of this review.

References

1. Moher D, Shamseer L, Clarke M, et al. Preferred reporting items for systematic review and meta-analysis protocols (PRISMA-P) 2015 statement. *Systematic reviews*. 2015;4(1):1.

2. Quayle KS, Jaffe DM, Kuppermann N, et al. Diagnostic testing for acute head injury in children: When are head computed tomography and skull radiographs indicated? *Pediatrics*. 1997;99(5):E11.

3. Kuppermann N, Holmes JF, Dayan PS, et al. Identification of children at very low risk of clinically-important brain injuries after head trauma: A prospective cohort study. *The Lancet*. 2009;374(9696):1160-1170.

4. Dunning J, Daly JP, Lomas J, Lecky F, Batchelor J, Mackway-Jones K. Derivation of the children's head injury algorithm for the prediction of important clinical events decision rule for head injury in children. *Arch Dis Child*. 2006;91(11):885-891.

5. Osmond MH, Klassen TP, Wells GA, et al. CATCH: A clinical decision rule for the use of computed tomography in children with minor head injury. *CMAJ*. 2010;182(4):341-348. doi: 10.1503/cmaj.091421 [doi].

6. Schutzman SA, Greenes DS. Pediatric minor head trauma. *Ann Emerg Med*. 2001;37(1):65-74.

7. Boran BO, Boran P, Barut N, Akgun C, Celikoglu E, Bozbuga M. Evaluation of mild head injury in a pediatric population. *Pediatr Neurosurg*. 2006;42(4):203-207. doi: 92355 [pii].

8. Shane SA, Fuchs SM. Skull fractures in infants and predictors of associated intracranial injury. *Pediatr Emerg Care*. 1997;13(3):198-203.

9. Miglioretti DL, Johnson E, Williams A, et al. The use of computed tomography in pediatrics and the associated radiation exposure and estimated cancer risk. *JAMA pediatrics*. 2013;167(8):700-707.

10. Brenner DJ, Elliston CD, Hall EJ, Berdon WE. Estimated risks of radiation-induced fatal cancer from pediatric CT. *Am J Roentgenol*. 2001;176(2):289-296.

11. Hennelly KE, Mannix R, Nigrovic LE, et al. Pediatric traumatic brain injury and radiation risks: A clinical decision analysis. *J Pediatr*. 2013;162(2):392-397. doi: 10.1016/j.jpeds.2012.07.018 [doi].

12. Meulepas JM, Ronckers CM, Smets AM, et al. Radiation exposure from pediatric CT scans and subsequent cancer risk in the netherlands. *JNCI: Journal of the National Cancer Institute*. 2018.

13. Moher D, Liberati A, Tetzlaff J, Altman DG. Preferred reporting items for systematic reviews and meta-analyses: The PRISMA statement. *Ann Intern Med*. 2009;151(4):264-269.

14. Whiting PF, Rutjes AW, Westwood ME, et al. QUADAS-2: A revised tool for the quality assessment of diagnostic accuracy studies. *Ann Intern Med*. 2011;155(8):529-536.

**Table 1:** Search syntax per search engine.
Ovid Medline:
("Head Injuries, Closed"/ OR head/ OR Skull Fractures/ OR skull/ OR (skull OR cranium OR cranial OR calvarium OR scalp OR skullcap OR head).ab,ti,kw.) AND (Ultrasonography/ OR Ultrasonics/ OR (echogra* OR ultraso* OR sonogra* OR pocus).ab,ti,kw.) AND (Fractures, Bone/ OR Skull Fractures/ OR (fracture*).ab,ti,kw.) AND (exp child/ OR adolescent/ OR exp infant/ OR Pediatrics/ OR pediatric hospital/ OR childhood injury/ OR (child* OR infan* OR adolescen* OR pediatr* OR paediatr*).ab,ti,kw.)
Embase:
('head injury'/de OR 'head'/de OR 'skull injury'/exp OR 'skull'/exp OR (skull OR cranium OR cranial OR calvarium OR scalp OR skullcap OR head):ab,ti,kw) AND ('echography'/de OR 'focused assessment with sonography for trauma'/de OR ultrasound/de OR (echogra* OR ultraso* OR sonogra* OR pocus):ab,ti,kw) AND ('fracture'/de OR 'skull fracture'/exp OR (fracture*):ab,ti,kw) AND (juvenile/exp OR pediatrics/exp OR 'pediatric hospital'/de OR 'childhood injury'/de OR (child* OR infan* OR adolescen* OR pediatr* OR paediatr*):ab,ti,kw)
Cochrane CENTRAL
((skull OR cranium OR cranial OR calvarium OR scalp OR skullcap OR head):ab,ti,kw) AND ((echogra* OR ultraso* OR sonogra* OR pocus):ab,ti,kw) AND ((fracture*):ab,ti,kw) AND ((child* OR infan* OR adolescen* OR pediatr* OR paediatr*):ab,ti,kw)
Web of science
TS=(((skull OR cranium OR cranial OR calvarium OR scalp OR skullcap OR head)) AND ((echogra* OR ultraso* OR sonogra* OR pocus)) AND ((fracture*)) AND ((child* OR infan* OR adolescen* OR pediatr* OR paediatr*)))
Google Scholar
skull|cranium|cranial|calvarium|scalp|skullcap|head echogram|echography|ultrasonography|ultrasound|sonogram fracture child| children|infant|adolescent|pediatric|paediatric|infants|adolescents|pediatrics|paediatrics
