# Supplementary material for: Evidence base for point-of-care ultrasound (POCUS) for diagnosis of skull fractures in children: a systematic review and meta-analysis
Source: Emerg Med J. 2020 Dec 3;39(1):30–6. doi: 10.1136/emermed-2020-209887 (PMC8717482; doi:10.1136/emermed-2020-209887)
Supplement: Supplementary data [file emermed-2020-209887supp002.pdf]

Table 1: Search syntax per search engine. Search performed on 17th of July 2020.

Ovid Medline:

("Head Injuries, Closed"/ OR head/ OR Skull Fractures/ OR skull/ OR (skull OR cranium OR cranial OR calvarium OR scalp OR skullcap OR head).ab,ti,kw.) AND (Ultrasonography/ OR Ultrasonics/ OR (echogra* OR ultraso* OR sonogra* OR pocus).ab,ti,kw.) AND (Fractures, Bone/ OR Skull Fractures/ OR (fracture*).ab,ti,kw.) AND (exp child/ OR adolescent/ OR exp infant/ OR Pediatrics/ OR pediatric hospital/ OR childhood injury/ OR (child* OR infan* OR adolescen* OR pediatr* OR paediatr*).ab,ti,kw.)

Embase:

('head injury'/de OR 'head'/de OR 'skull injury'/exp OR 'skull'/exp OR (skull OR cranium OR cranial OR calvarium OR scalp OR skullcap OR head):ab,ti,kw) AND ('echography'/de OR 'focused assessment with sonography for trauma'/de OR ultrasound/de OR (echogra* OR ultraso* OR sonogra* OR pocus):ab,ti,kw) AND ('fracture'/de OR 'skull fracture'/exp OR (fracture*):ab,ti,kw) AND (juvenile/exp OR pediatrics/exp OR 'pediatric hospital'/de OR 'childhood injury'/de OR (child* OR infan* OR adolescen* OR pediatr* OR paediatr*):ab,ti,kw)

Cochrane CENTRAL

((skull OR cranium OR cranial OR calvarium OR scalp OR skullcap OR head):ab,ti,kw) AND ((echogra* OR ultraso* OR sonogra* OR pocus):ab,ti,kw) AND ((fracture*):ab,ti,kw) AND ((child* OR infan* OR adolescen* OR pediatr* OR paediatr*):ab,ti,kw)

Web of science

TS=(((skull OR cranium OR cranial OR calvarium OR scalp OR skullcap OR head)) AND ((echogra* OR ultraso* OR sonogra* OR pocus)) AND ((fracture*)) AND ((child* OR infan* OR adolescen* OR pediatr* OR paediatr*)))

Google Scholar

skull|cranium|cranial|calvarium|scalp|skullcap|head
echogram|echography|ultrasonography|ultrasound|sonogram fracture child|
children|infant|adolescent|pediatric|paediatric|infants|adolescents|pediatrics|paediatrics
